# Inhibitory role of angiopoietin-like 4 for cancer progression in oropharyngeal squamous cell carcinoma

**DOI:** 10.3892/or.2026.9122

**Published:** 2026-04-20

**Authors:** Takuya Mikoshiba, Mariko Sekimizu, Nana Nakahara, Shin Saito, Keisuke Yoshihama, Ryoto Nagai, Takeyuki Kono, Hiroyuki Ozawa

**Affiliations:** Department of Otolaryngology, Head and Neck Surgery, Keio University School of Medicine, Tokyo 160-8582, Japan

**Keywords:** ANGPTL4, OPSCC, cancer progression, inhibitory role, prognosis

## Abstract

The mortality rate of head and neck squamous cell carcinoma (HNSCC), one of the most prevalent types of cancer, has remained unchanged despite advances in treatment strategies. Angiopoietin-like 4 (ANGPTL4) exhibits both pro- and anti-tumorigenic roles in various cancers depending on the tissue context. The present study investigated the role of ANGPTL4 expression in oropharyngeal squamous cell carcinoma (OPSCC). Data from 137 patients with OPSCC who underwent initial treatment were retrospectively analyzed. ANGPTL4 expression in tumor tissues was determined via immunohistochemistry. Survival outcomes [overall survival (OS) and disease-free survival (DFS)] and clinicopathological correlations were evaluated. Tumor cell proliferation was assessed using an CellTiter-Glo 2.0 luminescence-based cell viability assay, immunofluorescence staining, and reverse transcription-quantitative polymerase chain reaction following small interfering RNA-mediated knockdown in a HNSCC cell line. Gene set enrichment analysis (GSEA) was performed using data from The Cancer Genome Atlas. ANGPTL4 expression (≥7.7%) in patients with OPSCC was significantly associated with improved OS and DFS. Multivariate analysis confirmed that low ANGPTL4 expression was an independent prognostic factor for OS [hazard ratio (HR)=3.676, 95% confidence interval (CI)=1.678–8.056; P=0.001) and DFS (HR=2.959, 95% CI=1.533–5.713; P=0.001)]. FaDu cells with *ANGPTL4* knockdown demonstrated significantly increased proliferation compared with negative controls in the CellTiter-Glo 2.0 assay (P=0.010), accompanied by a significant increase in Ki-67 expression as revealed by immunofluorescence staining (P=0.021). The relative *ANGPTL4* mRNA expression levels decreased to 38%, whereas those of *MKI67* increased significantly. GSEA revealed significant enrichment of cell cycle progression signatures in cases with low *ANGPTL4* expression. ANGPTL4 expression was significantly associated with a favorable prognosis in OPSCC, and its knockdown increased proliferative activity in FaDu cells. Thus, ANGPTL4 may serve as a prognostic biomarker in OPSCC. Further *in vivo* studies are warranted to clarify causality and assess the therapeutic potential of *ANGPTL4* targeting in OPSCC.

## Introduction

Head and neck squamous cell carcinoma (HNSCC) is one of the most prevalent types of cancer, accounting for ~3.6% of all new cancer cases in the United States (1). Advances in the treatment of HNSCC have been made over the past few decades, but the mortality rate has remained essentially unchanged (2,3). HNSCC encompasses a diverse range of tumors, including squamous cell carcinoma (SCC) in the oral cavity, pharynx, larynx, nasal cavity and paranasal sinuses and salivary glands (4). Human papillomavirus (HPV) is a widely recognized risk factor for carcinogenesis and prognosis in patients with oropharyngeal squamous cell carcinoma (OPSCC). HPV-positive OPSCC is associated with a more favorable prognosis, whereas HPV-negative OPSCC is associated with a poor prognosis. Furthermore, high cell proliferation also emerges as a firmly established adverse prognostic factor. A meta-analysis revealed a correlation between a high proliferation index and lower survival in patients with HNSCC (5).

Angiopoietin-like 4 (ANGPTL4) is a member of the angiopoietin-related family, which has been reported to play a crucial role in regulating glucose and lipid metabolism (6). In the field of oncology, it has been previously demonstrated that ANGPTL4 has been reported in various cancers, including HNSCC (7). ANGPTL4 has been shown to play multiple roles in cancer progression, including tumor growth, anoikis resistance, angiogenesis, tumor invasion and metastasis. Most previous studies have described that the upregulation of ANGPTL4 is associated with the promotion of tumor growth, progression, angiogenesis, invasion and metastasis and reduces overall survival (OS) (8–14). By contrast, ANGPTL4 has also been reported to have an inhibitory function against tumor growth, angiogenesis, and vascular leakiness, prevents metastasis and is associated with an improved prognosis (15–18). Hsieh *et al* (19) reported that ANGPTL4 had both oncogenic and tumor-suppressing roles in urothelial carcinoma. These conflicting results indicate that ANGPTL4 could be both promotive and inhibitory for tumorigenesis, depending on tissue context and organ site. Few studies have reported on the roles of ANGPTL4 in HNSCC. In the present study, it was investigated whether ANGPTL4 expression is associated with pro- or anti-tumorigenic effects in OPSCC.

## Materials and methods

### Patient and tissue specimens

The data of 146 patients with OPSCC who underwent initial surgery or biopsy at the Department of Otolaryngology, Head and Neck Surgery, Keio University School of Medicine between April 2005 and September 2018 were retrospectively reviewed. A total of three patients who had received chemotherapy and one patient who had received radiotherapy for the head and neck region before the initial surgery or biopsy were excluded. A total of five patients who underwent biopsy only and were treated at other hospitals were also excluded. Consequently, 137 patients were enrolled in the present study. The cohort included 116 men and 21 women. Their median age was 64 years (range, 37–87 years). Their characteristics (age, sex, smoking and alcohol status, tumor subsite, TNM classification, pathologic characteristics, and follow-up examination findings) were extracted from their medical records. The TNM classification was determined based on the eighth edition of the American Joint Committee on Cancer staging manual (20). The histopathological diagnoses were based on the World Health Organization criteria (21). Tissue samples were obtained from the hospital tissue bank.

The treatment strategy for OPSCC in the present study was as follows. Early T-stage (T1 and T2) tumors were treated with transoral resection or radiotherapy alone. Advanced T-stage (T3 and T4) tumors were treated with concurrent chemoradiotherapy (chemotherapy: Cisplatin 80 mg/m^2^, administered 2–3 times every 3 weeks; radiotherapy: 2.0 Gy/fraction, administered five times a week for a total dose of 60–66 Gy) or radical tumor resection with functional reconstruction. The patients with multiple LN metastases underwent neck dissection before radiotherapy or concurrently with primary surgery. The patients with resectable locoregional recurrences or neck metastases detected during follow-up underwent additional resections immediately. They received chemotherapy as palliative treatment for persistent disease or distant metastases if they were amenable to treatment according to the NCCN guidelines (4).

### Immunohistochemistry (IHC)

The paraffin sections were sliced at 5 µm each, dewaxed in xylene, rehydrated in ethanol, and washed in water. Heat-induced antigen retrieval was performed using the Decloaking Chamber NxGen^®^ (Biocare Medical, LLC) with Dako Target Retrieval Solution (cat. no. S1700; Dako; Agilent Technologies, Inc.) at 95°C for 1 h. Endogenous peroxidase activity was quenched by treatment with 3% hydrogen peroxide solution for 10 min. Non-specific binding of the primary antibodies was blocked by treatment with normal goat serum (cat. no. IHR-8136; ImmunoBioScience/IBC) in phosphate-buffered saline (PBS) containing TWEEN^®^20 for 1 h at room temperature. The slides were stained with the primary antibody (1:500) overnight at 4°C. They were washed and incubated with SignalStain^®^ Boost IHC Reagent (HRP; Rabbit; cat. no. 8114; Cell Signaling Technology, Inc.) for 30 min at room temperature. Peroxidase activity was visualized using a DAB Substrate Kit (cat. no. SK-4100; Vector Laboratories, Inc.). The sections were counterstained and sealed with hematoxylin solution. The stained slides were imaged using a light microscope. The tissue images were imported, and the proportions of ANGPTL4-positive tumor cells were determined using Tissue Studio^®^ (Definiens). The tumorous area was selected as the region of interest for each slide. The following parameters were set: Hematoxylin threshold of 0.2, typical nucleus size of 23 µm^2^, maximum cell growth of 5, and classification of 0.15. ANGPTL4 expression was automatically calculated as the ratio of the number of ANGPTL4-positive tumor cells to the total number of tumor cells.

### Cell line

FaDu, Detroit 562, OSC-19, HSC-2 and HSC-4 (human HNSCC cell lines) were obtained from the American Type Culture Collection.

### Cell culture

The cell lines were cultured in Eagle's Minimum Essential Medium (Sigma-Aldrich; Merck KGaA) supplemented with 10% fetal bovine serum (FBS) and 1% penicillin-streptomycin (solution stabilized; Sigma-Aldrich; Merck KGaA). They were incubated in a humidified incubator at 37°C in a 5% carbon dioxide environment. The cells were sub-cultured continuously in accordance with the American Type Culture Collection protocol.

### Reverse transcription-quantitative polymerase chain reaction (RT-qPCR)

RNA was extracted using the RNeasy Mini Kit (Qiagen GmbH), and its quality was assessed using the Nanodrop 1000 (Thermo Fisher Scientific, Inc.). Complementary DNA synthesis was performed using the Superscript III First-Strand Synthesis System (Invitrogen; Thermo Fisher Scientific, Inc.) according to the manufacturer's instructions. RT-qPCR was conducted using the StepOnePlus Real-Time PCR system and software (Applied Biosystems; Thermo Fisher Scientific, Inc.) following the manufacturer's protocol. The primers and probes were procured from Applied Biosystems (TaqMan^®^ Gene Expression Assays) and had the following IDs: *ANGPTL4* (Hs01101127_m1), *ACTB* (Hs01060665_g1), *MKI67* (Hs04260396_g1), *BAX* (Hs0018269_m1), *BCL2* (Hs00608023_m1), and *CASP3* (Hs00234387_m1). According to the manufacturer's product information, the sequences for these pre-designed assays are not publicly disclosed. PCR amplification involved initial denaturation at 95°C for 20 sec, followed by 40 cycles of denaturation at 95°C for 3 sec and annealing at 60°C for 30 sec. The relative mRNA expression levels were determined using the 2^−ΔΔCq^ method and were compared with those of *ACTB*, which served as the endogenous control (22).

### Small interfering RNA (siRNA) knockdown

The FaDu cells were seeded in a 6-well dish at a density of 25,000 cells/ml and incubated in a medium containing 10% FBS for 24 h. The medium was changed, and the cells were transfected with 30 nM siRNA targeting *ANGPTL4* (cat. no. NM_001039667; ID: SASI_Hs01_00144609; Sigma-Aldrich; Merck KGaA) and negative control (MISSION siRNA Universal Negative Control #1; Sigma-Aldrich; Merck KGaA) using 4 µl of Lipofectamine 2000 Transfection Reagent (Thermo Fisher Scientific, Inc.) in 200 µl of Opti-MEM medium (Thermo Fisher Scientific, Inc.) for 30 min at room temperature. The Opti-MEM medium was removed, and 2 ml of fresh culture medium was added. The cells were incubated for an additional 24 h, scraped, and collected for analysis. The efficiency of *ANGPTL4* knockdown was evaluated using RT-qPCR. According to the manufacturer's product information, the sequences of the siRNAs used in the present study are not publicly disclosed.

In addition to FaDu cells, *ANGPTL4* knockdown experiments were attempted in other HNSCC cell lines, including Detroit 562, OSC-19, HSC-2 and HSC-4, using the same siRNA sequence and transfection protocols.

### Cell proliferation assay (CellTiter-Glo 2.0 luminescence assay)

The FaDu cells were seeded in a 96-well plate (3.000 cells per well) and incubated overnight after transfection. Their viability was subsequently assessed using a CellTiter-Glo 2.0 luminescence-based assay kit (cat. no. G9241; Promega Corporation). The results were normalized to those of the negative control (set as 1.0).

### Immunofluorescence staining

The FaDu cells were seeded in slide chambers (CHAMBER SLIDEII IWAKI, http://iwaki.atgc.co.jp) after *ANGPTL4* knockdown for immunofluorescence analyses of ANGPTL4 and Ki-67. The cells were washed extensively with PBS and fixed with 4% paraformaldehyde for 15 min at room temperature. The samples were washed with PBS and blocked with 10% normal goat serum in PBS for 1 h. The cells were incubated with the primary antibodies overnight at 4°C. They were subsequently incubated with Alexa Fluor Plus 555- and 488-conjugated secondary antibodies (cat. nos. A32732 and A32723; Thermo Fisher Scientific, Inc.) against ANGPTL4 and Ki-67, respectively, and observed 24 h after transfection. Hoechst 33258 (Sigma-Aldrich) was used for nuclear staining. The numbers of ANGPTL4- and Ki-67-positive cells were determined based on counts in four randomly selected areas at ×10 magnification using a BZ-X710 fluorescence microscope (Keyence Corporation).

### Antibody

The primary antibodies used for immunostaining were anti-ANGPTL4 (rabbit; 1:500; cat. no. ab115798; Abcam) for immunofluorescence staining and IHC and anti-Ki-67 (mouse; 1:100; cat. no. ab245113; Abcam) for immunofluorescence staining.

### The cancer genome atlas (TCGA)

RNA sequencing and corresponding clinical data for HNSCC were obtained from TCGA via the University of California Santa Cruz Cancer Browser (https://xenabrowser.net/). A total of 78 patients with primary OPSCC and corresponding clinical data from the cohort labeled ‘GDC TCGA Head and Neck Cancer’ were included. The patients were divided into high and low groups based on their *ANGPTL4* expression levels (FPKM-UQ). The prognoses of 128 and 116 patients with tongue and laryngeal SCCs, respectively, were also evaluated. The *ANGPTL4* expression data for 520 patients with HNSCC were extracted based on the subsites. Gene set enrichment analysis (GSEA) was performed based on the *ANGPTL4* expression to explore its biological role in OPSCC. GSEA was performed using the Hallmark gene set from the Molecular Signatures Database (version 7.5.1, http://www.gsea-msigdb.org/gsea/msigdb/index.jsp). The enrichment score, normalized enrichment score, nominal P-value and false discovery rate (FDR) were determined using UCSC Xena (https://xenabrowser.net/). Statistical significance was set at FDR <0.05. The differentially expressed genes (DEGs) were extracted based on a fold change of >2 or <-2 and adjusted P-value of <0.05.

### Statistical analysis

The five-year OS and disease-free survival (DFS) rates of the patients with OPSCC were determined using the Kaplan-Meier method, and survival curves were compared using the log-rank test. When survival curves crossed, weighted tests (Breslow and Tarone-Ware) were additionally performed as sensitivity analyses. The survival durations were calculated from the date of initial treatment to the date of the event or the latest follow-up. The variables included were age, sex, smoking and alcohol status, T classification, N classification, M classification, TNM stage, p16 status, initial definitive therapy and ANGPTL4 status. To determine the optimal cut-off value for ANGPTL4 expression, receiver operating characteristic (ROC) curve analysis was performed using the OS. The cut-off value was defined as the point that maximized Youden's index. The ROC curve is provided in Fig. S1. To evaluate the robustness of the selected cut-off value, sensitivity analyses were performed using alternative thresholds for ANGPTL4 expression. Tumors were reclassified using the median expression level, as well as fixed cut-off values of 10 and 15%. The OS and DFS of the patients in the subgroups were compared using the log-rank test during the univariate analysis. The factors that were significant in the univariate analysis were further analyzed using multivariate analysis. This was performed using a Cox proportional hazards model with backward elimination. The correlations between the variables analyzed in the multivariate analysis were examined using Pearson's correlation coefficient to avoid multicollinearity. The relationships between ANGPTL4 and the other variables were evaluated. The distributions of the categorical variables for ANGPTL4 and the other variables were compared using the chi-squared test. In addition, survival analyses were performed stratified by p16 status to evaluate the prognostic impact of ANGPTL4 within each subgroup. Furthermore, sensitivity analyses were performed using the multivariable Cox models in patients who underwent definitive therapy. Associations between continuous variables were analyzed using unpaired Student's t-test. The differences in ANGPTL4 expression across the subsites were determined using one-way analysis of variance. Post hoc tests were not performed. Statistical analyses were performed using SPSS version 27 for Mac (IBM Corp.). P<0.05 was considered to indicate a statistically significant difference.

## Results

### High ANGPTL4 expression is associated with a favorable prognosis in patients with OPSCC

**Figure 1.**
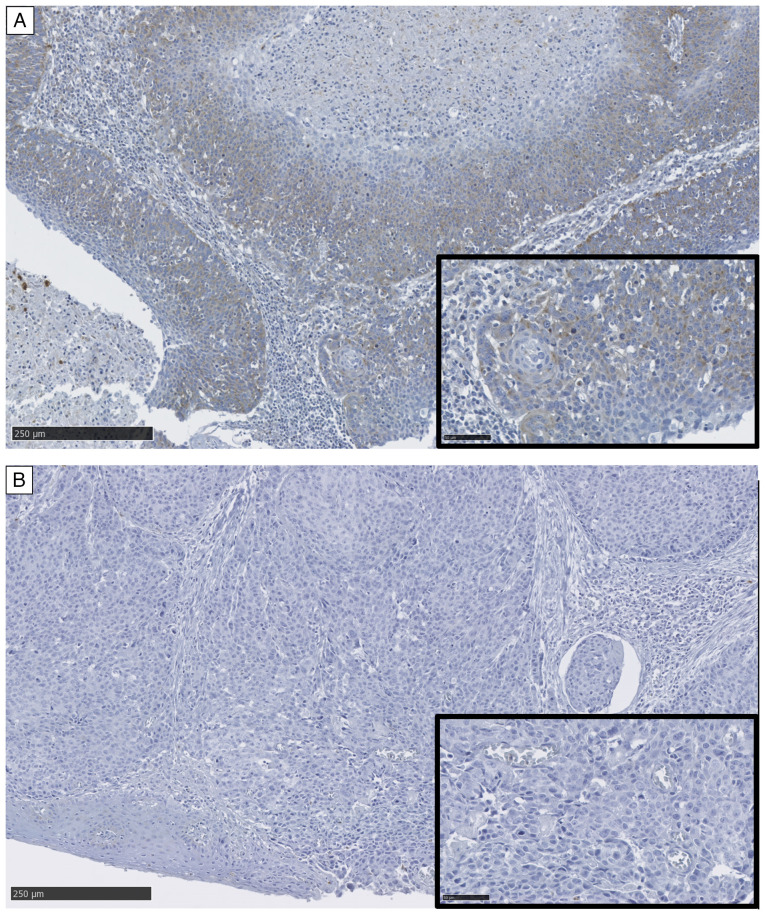
Immunohistochemical staining of oropharyngeal squamous cell carcinoma. Representative cases with (A) positive and (B) negative staining of angiopoietin-like 4 are shown (lower magnification, ×100; scale bar, 250 µm; higher magnification, ×400; scale bar, 50 µm).

**Figure 2.**
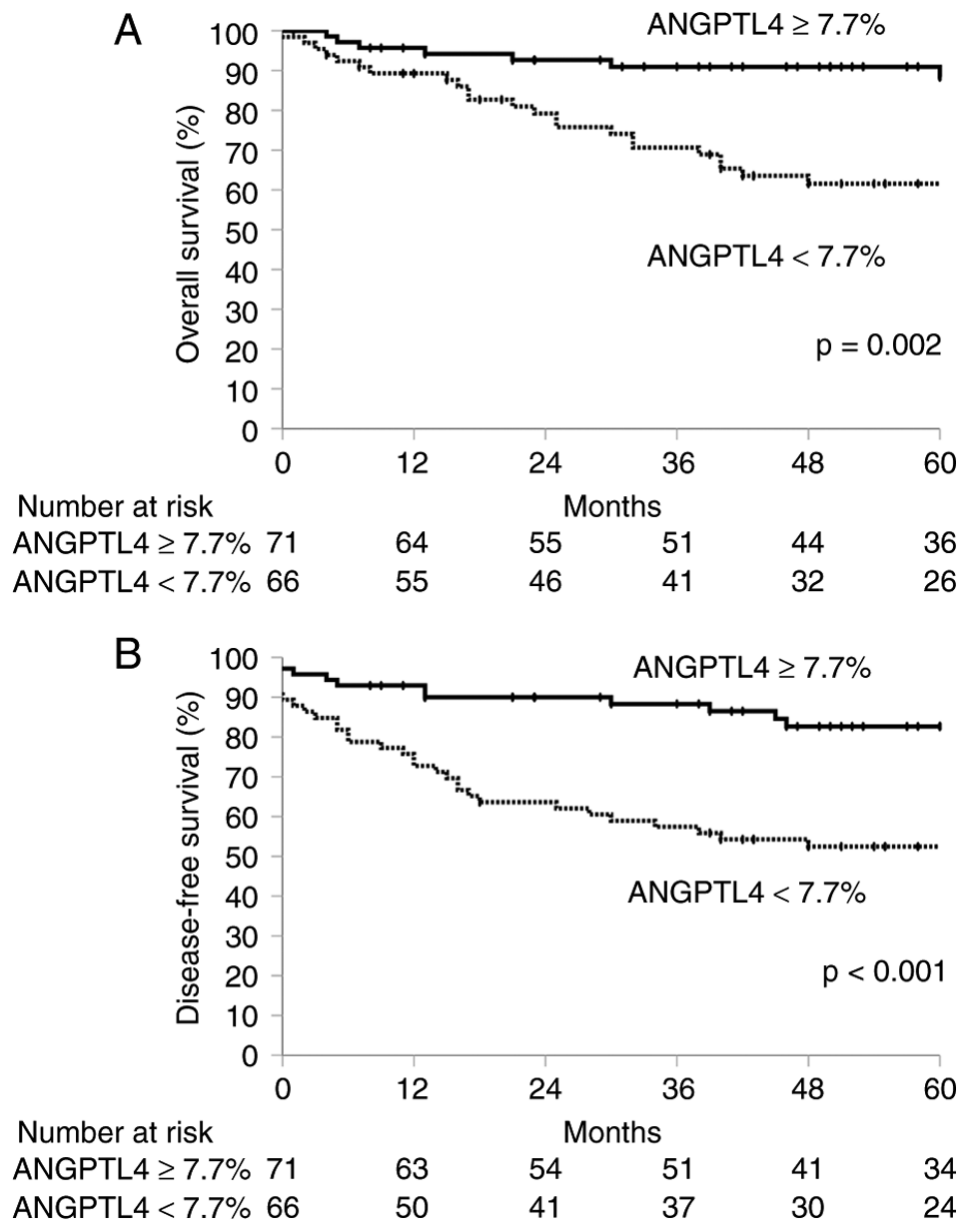
(A) Overall survival and (B) disease-free survival of patients with oropharyngeal squamous cell carcinoma based on ANGPTL4. ANGPTL4, angiopoietin-like 4.

The characteristics of the 137 patients with OPSCC included in the present study are presented in Table I. In total, 40 had locally advanced disease (T3 and T4), 101 had cervical lymph node metastases, 2 had distant metastases, and 39 had advanced TNM stages (III and IV). A total of ~80% of the patients were positive for p16 staining. The IHC staining for ANGPTL4 is shown in Fig. 1. Totally, 71 and 66 patients had positive and negative staining for ANGPTL4, respectively. The 5-year OS and DFS rates were 75.1 and 67.6%, respectively. The median follow-up duration was 52 months (range, 1–171 months). The univariate analyses of OS and DFS based on the clinicopathological factors are summarized in Table II. T classification (T3 and T4), M classification (M1), TNM stage (III and IV) and ANGPTL4 (<7.7%) were significantly associated with poorer OS (P=0.001, P<0.001, P=0.034 and P=0.002, respectively). The subsite (anterior wall), T classification (T3 and T4), M classification (M1), TNM stage (III and IV) and ANGPTL4 (<7.7%) were significant predictors of worse DFS in patients with OPSCC (P=0.007, P<0.001, P<0.001, P=0.007, and P<0.001, respectively). Multivariate analysis revealed that T classification [hazard ratio (HR)=3.209; 95% confidence interval (CI), 1.605–6.415; P<0.001], M classification (HR=94.613; 95% CI, 15.385–581.837; P<0.001) and ANGPTL4 (HR=3.676; 95% CI, 1.678–8.056; P=0.001) were independent prognostic factors for OS. The subsite (HR=2.161; 95% CI, 1.183–3.947; P=0.012), T classification (HR=3.029; 95% CI, 1.652–5.550; P<0.001), M classification (HR=15.081; 95% CI, 3.083–73.771; P<0.001) and ANGPTL4 (HR=2.959; 95% CI, 1.533–5.713; P=0.001) were independent prognostic factors for DFS in patients with OPSCC (Table III). The Kaplan-Meier curves for OS and DFS stratified by ANGPTL4 expression are shown in Fig. 2. The 5-year OS rates for patients who tested positive and negative for ANGPTL4 expression were 88.4 and 61.6%, respectively (P=0.002). The corresponding 5-year DFS rates were 82.7 and 52.5%, respectively (P<0.001). Sensitivity analyses using alternative cut-off values including median ANGPTL4 expression level, 10%, and 15% demonstrated consistent results (Table SI). In addition, baseline characteristics stratified by p16 status are summarized in Table SII. High ANGPTL4 expression was associated with improved OS and DFS in the p16-positive subgroup, whereas no significant prognostic impact was observed in the p16-negative subgroup (Table SIII). In sensitivity analyses restricted to patients who received definitive therapy, additional adjustment for p16 status (model 1) and for initial definitive therapy (model 2) did not alter the prognostic association of ANGPTL4 with the OS and DFS (Table SIV).

The correlations between ANGPTL4 expression and clinicopathological factors in OPSCC are provided in Table IV. No significant correlations were observed between ANGPTL4 expression and age, sex, subsite, stage, or p16 expression.

### ANGPTL4 expression inhibits cell proliferation in the FaDu cell lines

**Figure 3.**
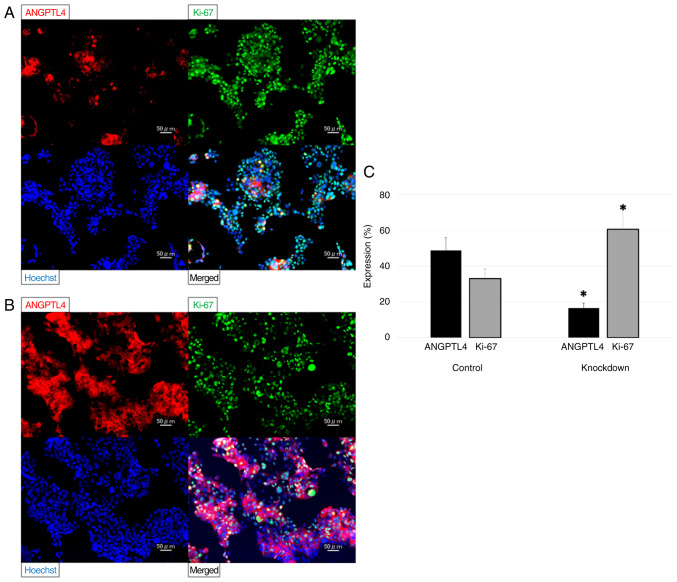
Fluorescence intensities of anti-angiopoietin-like 4 (red), anti-Ki-67 (green), and Hoechst (blue) for the FaDu cells. (A-C) The fluorescence intensities of the FaDu cells transfected with angiopoietin-like 4 were significantly different from those of the control. *P<0.05.

The FaDu cells were transfected with siRNA targeting *ANGPTL4*, and their proliferation was evaluated compared with the negative controls. Their relative mRNA expression levels were determined using RT-qPCR. The relative mRNA expression level of *ANGPTL4* decreased to 38%. The results of immunofluorescence staining are provided in Fig. 3. The knockdown cells had significantly reduced ANGPTL4 expression and significantly increased Ki-67 expression compared with the negative controls (P=0.021 and 0.021, respectively).

**Figure 4.**
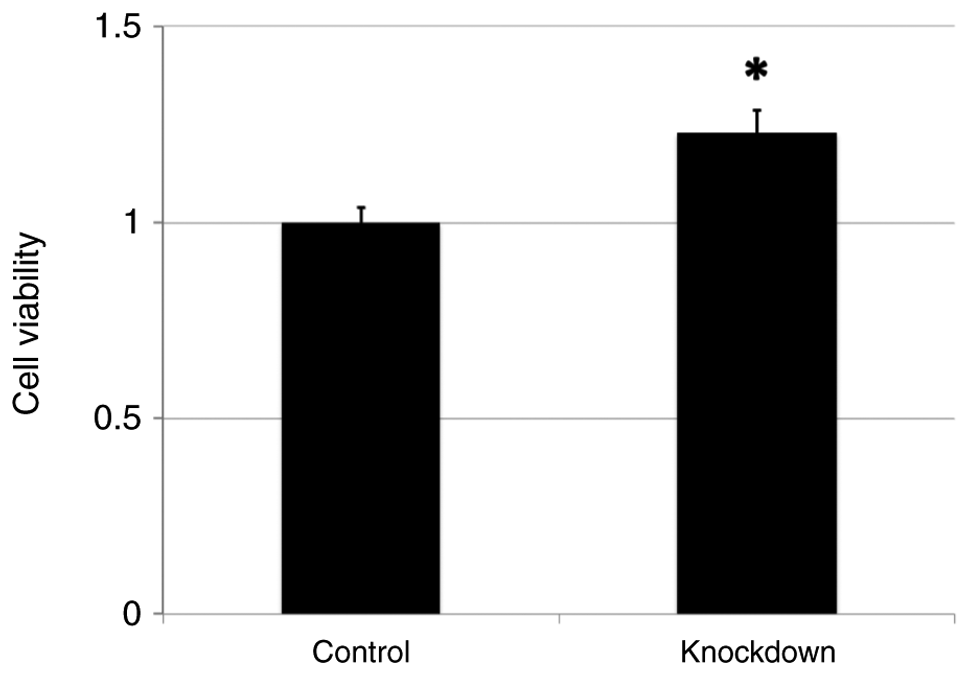
CellTiter-Glo 2.0 luminescence assay revealed a significant increase in cell viability following angiopoietin-like 4 knockdown relative to the controls (set as 1.0) (P=0.010). *P<0.05.

The FaDu cells with ANGPTL4 knockdown showed significantly increased proliferation compared with the negative controls (P=0.010) (Fig. 4).

*ANGPTL4* knockdown was attempted in additional HNSCC cell lines (Detroit 562, OSC-19, HSC-2 and HSC-4); however, robust and reproducible knockdown could not be achieved. Therefore, functional analyses were performed only in FaDu cells.

### Knockdown of ANGPTL4 expression enhances the cell proliferation signal in FaDu cells

**Figure 5.**
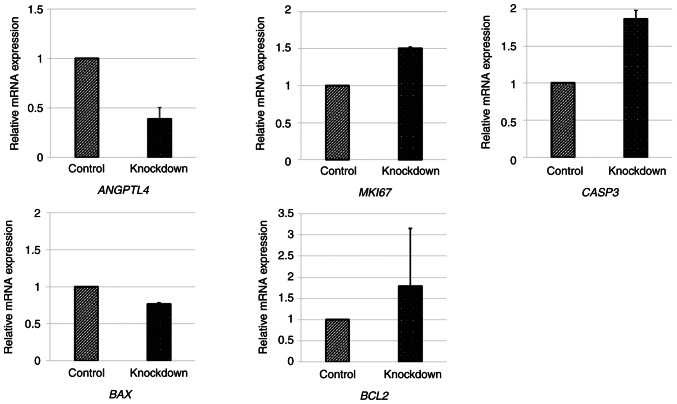
Relative gene expression levels of various mRNAs in the FaDu cells transfected with *angiopoietin-like 4* small interfering RNA were determined by reverse transcription-quantitative PCR. The *angiopoietin-like 4* level decreased by 38%, and *MKI67* expression increased significantly. The expression of *BAX* decreased, and that of *BCL2* increased. *CASP3* expression also increased in the angiopoietin-like 4 knockdown cells.

The relative gene expression levels of various mRNAs in the FaDu cells transfected with *ANGPTL4* siRNA were determined via RT-qPCR (Fig. 5). The relative mRNA expression levels of *ANGPTL4* decreased to 38%, and those of *MKI67* increased significantly. The expression of apoptosis-related genes was also evaluated. The expression of *BAX* decreased, and that of *BCL2* increased. In addition, the expression of *CASP3* increased in the *ANGPTL4* knockdown cells.

### ANGPTL4 expression has different prognostic implications for the subsites of HNSCC based on TCGA data

**Figure 6.**
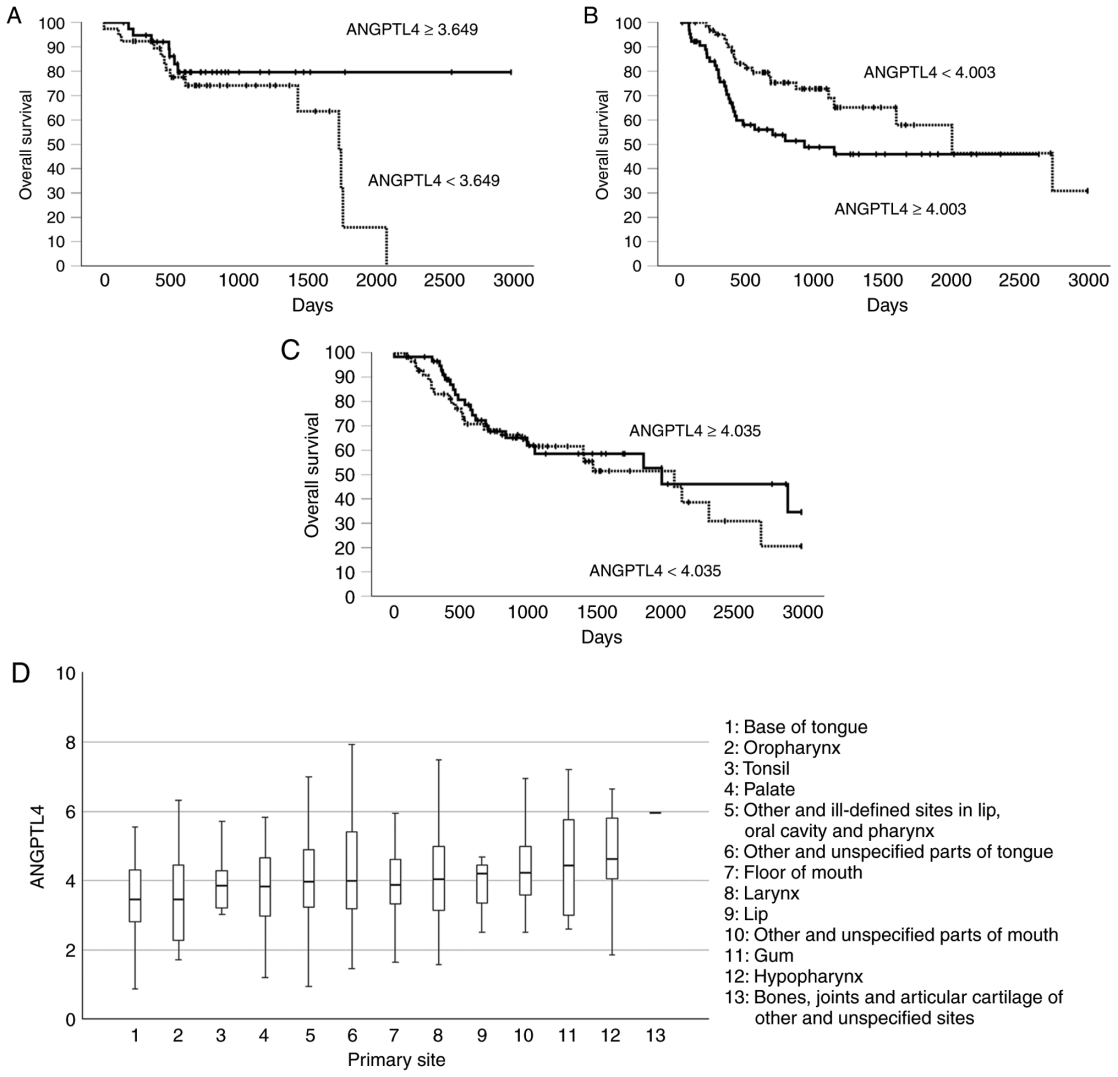
Correlations between ANGPTL4 expression and overall survival based on The Cancer Genome Atlas data for (A) oropharyngeal, (B) tongue and (C) laryngeal squamous cell carcinoma. The median FPKM-UQ value for ANGPTL4 was used as the cut-off to divide the groups into high and low. (D) The mean ANGPTL4 expression in head and neck squamous cell carcinoma was significantly different (P=0.042). ANGPTL4, angiopoietin-like 4.

The correlation between *ANGPTL4* expression and prognosis was analyzed using TCGA data. High *ANGPTL4* expression was associated with a tendency towards improved prognosis in 78 patients with OPSCC, but the difference was not statistically significant (P=0.09; Fig. 6A). The group with high *ANGPTL4* expression had significantly worse OS than the group with low expression in tongue SCC (log-rank, P=0.02; Breslow, P=0.003; Tarone-Ware, P=0.005; Fig. 6B). No significant differences in OS were detected between the patients with laryngeal SCC with high and low *ANGPTL4* expressions (log-rank, P=0.45; Breslow, P=0.47; Tarone-Ware, P=0.49; Fig. 6C). The mean *ANGPTL4* expression differed significantly across HNSCC subsites (P=0.042; Fig. 6D). The OPSCCs, including those of the base of the tongue, oropharynx and tonsils, had lower *ANGPTL4* expression levels than those of other subsites.

### ANGPTL4 expression is associated with a cell proliferation signature in the GSEA

GSEA revealed significant enrichment of the HALLMARK_E2F_TARGETS and HALLMARK_G2M_CHECKPOINT gene sets in the *ANGPTL4*-low expression group (NES=6.86 and 4.42, respectively; FDR <0.001 for both) (Table V). The HALLMARK_TNFA_SIGNALING_VIA_NFKB, HALLMARK_INTERFERON_GAMMA_RESPONSE, and HALLMARK_INFLAMMATORY_RESPONSE gene sets were significantly depleted in the *ANGPTL4*-low expression group. *NDRG1* and *CDH3* were identified as DEGs based on their fold changes (>2 or <-2) and adjusted P-values (<0.05) (Table VI).

## Discussion

Previous findings have revealed a critical role for ANGPTL4 in cancer growth and progression, angiogenesis, metastasis and anoikis resistance. ANGPTL4 has diverse roles of pro- and anti-tumorigenesis in different cancers (7), but its roles in HNSCC have been reported in a few studies (8,9). A previous study reported that EGF-induced ANGPTL4 plays a role in the regulation and progression of cancer metastasis (8); however, its inhibitory roles for tumor progression have not been fully elucidated. The findings of the present study suggest an inhibitory association of ANGPTL4 with tumor progression in OPSCC, which are supported by clinical outcomes and complementary *in vitro* and TCGA-based analyses. The present study revealed that higher ANGPTL4 expression (≥7.7%) in patients with OPSCC was significantly associated with improved OS and DFS. This finding suggested that ANGPTL4 could act as a tumor suppressor in OPSCC. Several studies have previously reported the correlation between the expression level of ANGPTL4 and survival rates in various cancers. Similar results were reported for gastric carcinoma (16), hepatocellular carcinoma (17) and triple-negative breast carcinoma (18). There have also been studies of no correlation between the expression of ANGPTL4 and prognoses of urothelial (19) and colorectal (23) carcinomas. Some studies reported that high ANGPTL4 expression was associated with poor prognoses of cervical (14), colorectal (10) and tongue (13) carcinomas. These variations suggest that the role of ANGPTL4 can differ with histology or tumor origin. The current analysis using TCGA data indicated that the expression and prognostic impact of *ANGPTL4* differed across the subsites of HNSCC, with poorer survival in tongue SCC, whereas no significant association was observed in laryngeal SCC. These subsite-specific patterns may reflect differences in etiologic exposures, oncogenic drivers and tumor microenvironmental states across anatomic locations, as genomic, transcriptional and microenvironmental heterogeneity has been reported among HPV-negative HNSCC arising from different subsites (24). In addition, ANGPTL4 is increasingly recognized as a context-dependent secreted factor whose functions can diverge based on the tumor type and microenvironment, and may be proteolytically processed into different forms that may have distinct biological functions (7,25). Accordingly, the clinical significance of ANGPTL4 may not be uniform across HNSCC and may require subsite-specific validation as a prognostic biomarker. On the other hand, a plausible explanation for the discrepancy with prior HNSCC studies (8,9) is that ANGPTL4 may exert distinct functions depending on the anatomic subsite and molecular context. In addition, HPV-positive OPSCC represents a distinct subtype, and HPV status may modify the clinical relevance of ANGPTL4 expression. Consistent with this, the current stratified analyses by p16 status demonstrated that the favorable prognosis of high ANGPTL4 expression was evident in the p16-positive subgroup, whereas no significant association was observed in the p16-negative subgroup. Notably, the TCGA-based GSEA revealed that low ANGPTL4 expression was associated with enrichment of cell cycle-related gene sets. These observations raise the hypothesis that the prognostic relevance of ANGPTL4 may be more pronounced in HPV-related tumors with deregulated checkpoints; however, this interpretation remains hypothesis-generating and warrants further validation in larger cohorts and mechanistic studies. On the other hand, the results of the present study demonstrated a favorable prognosis, which differed from those of prior studies describing a promotive role of ANGPTL4 in HNSCC (8,9). These studies primarily used metastasis-related *in vitro* assays (for example, anoikis resistance and invasion/migration) under specific stimuli, whereas the present study focused on clinical prognosis in OPSCC and proliferation-related phenotypes. Therefore, these context-dependent differences may account for the apparent discrepancy with prior studies.

The present study demonstrated that the downregulation of ANGPTL4 significantly increased the expression level of Ki-67 in immunofluorescence staining with the increase of *MKI67* mRNA expression and promoted cell proliferation in the CellTiter-Glo 2.0 assay. These findings suggested that ANGPTL4 can have an inhibitory role in cell proliferation in the HNSCC cell line FaDu. Previous studies have reported that ANGPTL4 has both promoting and inhibiting roles in cell proliferation in various cancers. Ito *et al* (15) reported that ANGPTL4 inhibited cell proliferation *in vitro* and *in vivo* by regulating angiogenesis and vascular leakiness. Ng *et al* (17) reported several mechanisms of ANGPTL4 in suppressing tumor progression, invasion and metastasis of HCC. Overexpression of ANGPTL4 suppressed tumor growth by enhancing tumor cell apoptosis, indicating that suppression of ANGPTL4 in HCC may be a way to escape from apoptosis (17). In addition, treatment with ANGPTL4-overexpressing adenovirus via portal vein significantly suppressed the formation of new vessels in the tumor by repressing the expression of vascular endothelial growth factor and suppressing the activation of Raf-MEK-Erk signaling pathway, suggesting an anti-angiogenic effect of ANGPTL4 on HCC. These *in vitro* findings were consistent with an antiproliferative association of ANGPTL4. However, the current functional assessments were limited to proliferation endpoints (CellTiter-Glo 2.0 assay and Ki-67), and other malignant phenotypes, including cell cycle progression, apoptosis, migration and invasion, were not examined. Furthermore, these functional observations were derived from siRNA-mediated knockdown without complementary rescue or overexpression experiments; therefore, off-target effects cannot be fully excluded. On the other hand, RT-qPCR revealed altered expression of apoptosis-related genes after *ANGPTL4* knockdown in FaDu cells. However, because apoptosis was not directly assessed using dedicated functional assays, these RT-qPCR findings should be interpreted as hypothesis-generating rather than as functional evidence of altered apoptosis. In OPSCC, further studies are needed to clarify the mechanism of the inhibitory role of ANGPTL4 for tumor cell proliferation.

GSEA using TCGA data revealed that both the HALLMARK_E2F_TARGETS and HALLMARK_G2M_CHECKPOINT pathways were significantly enriched in the low *ANGPTL4* group in OPSCC. These gene sets represent crucial regulators of cell cycle progression, particularly in the transition through the G1/S phase (E2F targets) and the G2/M checkpoint (26,27). High E2F activity increases the expression of genes involved in DNA replication and cell cycle progression, directly promoting proliferation (26). HALLMARK_G2M_CHECKPOINT includes genes that control the G2/M transition, ensuring cells only divide when DNA is correctly replicated. Activation of this checkpoint signature reflects increased cell cycle activity and is often observed in rapidly proliferating tumor cells (27). The present results of GSEA suggest that low ANGPTL4 expression is associated with enrichment of cell cycle-related gene sets in OPSCC. Because these transcriptomic findings are correlative, the mechanistic interpretation should be considered hypothesis-generating and will require dedicated validation, including flow cytometric cell-cycle analysis and protein-level assessment of key regulators. Furthermore, GSEA showed that inflammation- and immune-related signatures such as HALLMARK_TNFA_SIGNALING_VIA_NFKB,HALLMARK_INTERFERON_GAMMA_RESPONSE, and HALLMARK_INFLAMMATORY_RESPONSE were strongly suppressed. It is hypothesized that the decreased ANGPTL4 expression may be involved in the suppression of inflammatory signaling and may have some effect on the tumor microenvironment.

In the analysis of DEGs, *CDH3* and *NDRG1* were significantly upregulated in the high *ANGPTL4* expression group. CDH3 is a classical cadherin that plays a crucial role in maintaining epithelial cell-cell adhesion, contributing to the integrity of epithelial structures in normal tissues (28). In HNSCC, the high CDH3 expression was associated with poor prognosis and an advanced T stage (29,30). On the other hand, *NDRG1* is recognized as a tumor suppressor, which was reported to be associated with epithelial-mesenchymal transformation (31,32). A decrease in NDRG1 expression is associated with the promotion of tumor proliferation (33). In oral SCC, knockdown of *NDRG1* using short hairpin RNA significantly promoted cell proliferation, while overexpression of *NDRG1* caused cell cycle arrest at the S phase and suppressed proliferation. This suggested that NDRG1 acts to inhibit cell proliferation (33). In addition, knockdown of *NDRG1* enhanced cell proliferation, migration and invasion in nasopharyngeal carcinoma and increased tumor formation in mice (32). The significant reduction of these two genes (*NDRG1* and *CDH3*) in the low *ANGPTL4* expression group using TCGA data suggests that the expression of these genes is closely related to the impact of ANGPTL4 on tumor proliferation. To further analyze the function of the *ANGPTL4*, a larger case study is required.

The present study has certain limitations. First, the IHC analysis was retrospective and conducted at a single institution. Second, the cutoff value for ANGPTL4 positivity in IHC was relatively low. The evaluation was performed by a single evaluator, and formal reproducibility metrics (for example, Cohen's kappa) were not assessed; therefore, scoring variability cannot be excluded. Third, functional validation was limited to a single cell line, which was not derived from OPSCC and may not fully recapitulate OPSCC biology. In addition, *in vivo* experiments were not performed; therefore, the present study was unable to establish a causal role of ANGPTL4 in tumor growth. Further studies are needed to elucidate the mechanisms by which ANGPTL4 regulates cancer development.

The present study demonstrated significant associations of ANGPTL4 expression with prognosis in patients with OPSCC and showed that ANGPTL4 inhibited cell proliferation in the HNSCC cell line FaDu. ANGPTL4 may serve as a prognostic biomarker in OPSCC. Further *in vivo* studies are warranted to establish causality and clarify its clinical relevance.

## Supplementary Material

Supporting Data

Supporting Data

## Figures and Tables

**Table I.** Patient characteristics.

Variables	Cases (n=137)	Percentage, %
Age		
Mean (SD)	64 (10)	
Sex		
Male/Female	116/21	85/15
Smoking		
Yes/No	112/25	82/18
Alcohol		
Yes/No	104/33	76/24
Subsite		
Anterior wall	38	28
Lateral wall	83	61
Posterior wall	7	5
Superior wall	9	7
T classification		
T0/T1/T2/T3/T4	1/40/56/21/19	1/29/41/15/14
N classification		
N0/N1/N2/N3	36/71/27/3	26/52/20/2
M classification		
M0/M1	135/2	99/1
TNM stage		
0/I/II/III/IV	1/68/29/23/16	1/50/21/17/12
p16		
Positive/Negative	109/28	80/20
ANGPTL4		
≥7.7%/<7.7%	71/66	52/48

ANGPTL4, angiopoietin-like 4; SD, standard deviation.

**Table II.** Univariate analyses of prognostic factors for OS and DFS of patients with oropharyngeal carcinoma.

Variables	Cases	5-year OS (%)	P-value	5-year DFS (%)	P-value
Overall	137	75.1		67.6	
Age			0.685		0.063
<65	69	75.3		72.7	
≥65	68	75.8		62.5	
Sex			0.911		0.774
Male	116	75.6		67.5	
Female	21	71.3		67.1	
Smoking			0.400		0.320
Yes	112	73.1		64.9	
No	25	83.6		79.6	
Alcohol			0.380		0.199
Yes	104	72.5		65.0	
No	33	83.6		76.4	
Subsite			0.056		0.007
Anterior wall	38	65.1		50.7	
Others	99	79.1		74.3	
T classification			0.001		<0.001
0,1,2	97	81.9		77.1	
3,4	40	57.1		43.6	
N classification			0.692		0.944
0	36	72.2		65.2	
1,2,3	101	76.0		68.4	
M classification			<0.001		<0.001
0	135	76.2		68.6	
1	2	0		0	
TNM stage			0.034		0.007
I, II	98	78.7		73.7	
III, IV	39	65.3		51.5	
p16			0.677		0.765
Positive	109	74.0		67.8	
Negative	28	79.4		66.3	
Initial definitive therapy			0.910		0.275
Surgery	75	77.3		74.7	
Radiation or chemoradiation	53	79.2		67.9	
ANGPTL4			0.002		<0.001
≥7.7%	71	88.4		82.7	
<7.7%	66	61.6		52.5	

A total of 9 patients who did not undergo definitive therapy were excluded from the ‘Initial definitive therapy’ category. ANGPTL4, angiopoietin-like 4; DFS, disease-free survival; OS, overall survival.

**Table III.** Multivariate analysis of prognostic factors for OS and DFS of patients with oropharyngeal carcinoma.

	OS			DFS		
		
Variables	HR	95% CI	P-value	HR	95% CI	P-value
Subsite (Anterior wall)	-	-	-	2.161	1.183–3.947	0.012
T classification (3, 4)	3.209	1.605–6.415	<0.001	3.029	1.652–5.550	<0.001
M classification (1)	94.613	15.385–581.837	<0.001	15.081	3.083–73.771	<0.001
TNM stage (III, IV)	1.093	0.467–2.557	0.837	1.153	0.537–2.476	0.715
ANGPTL4 (<7.7%)	3.676	1.678–8.056	0.001	2.959	1.533–5.713	0.001

ANGPTL4, angiopoietin-like 4; DFS, disease free survival; OS, overall survival; HR, hazard ratio; CI, confidence interval.

**Table IV.** Univariate analysis of outcomes stratified by ANGPTL4 expression.

	ANGPTL4		
		
Variables	≥7.7%	<7.7%	P-value
Overall	71	66	
Age			0.795
<65	35	34	
≥65	36	32	
Sex			0.956
Male	60	56	
Female	11	10	
Subsite			0.518
Anterior wall	18	20	
Others	53	46	
T classification			0.161
1,2	54	43	
3,4	17	23	
N classification			0.602
0	20	16	
1,2,3	51	50	
M classification			0.733
0	70	65	
1	1	1	
TNM stage			0.224
I, II	54	44	
III, IV	17	22	
p16			0.828
Positive	57	52	
Negative	14	14	

ANGPTL4, angiopoietin-like 4.

**Table V.** Result of Gene Set Enrichment Analysis of in low *ANGPTL4* expression using The Cancer Genome Atlas data.

Term	ES	NES	P-value	FDR
HALLMARK_E2F_TARGETS	0.452643176	5.244648544	1.57×10^−7^	7.12×10^−7^
HALLMARK_G2M_CHECKPOINT	0.338413055	3.065174051	2.18×10^−3^	5.44×10^−3^
HALLMARK_INTERFERON_GAMMA_RESPONSE	−0.67230996	−8.693683244	3.51×10^−18^	1.75×10^−16^
HALLMARK_TNFA_SIGNALING_VIA_NFKB	−0.658426312	−8.483918737	2.18×10^−17^	5.44×10^−16^
HALLMARK_HYPOXIA	−0.615556414	−7.657331309	1.90×10^−14^	3.16×10^−13^
HALLMARK_INFLAMMATORY_RESPONSE	−0.722841711	−7.488593625	6.96×10^−14^	8.70×10^−13^
HALLMARK_INTERFERON_ALPHA_RESPONSE	−0.71989998	−7.157175996	8.24×10^−13^	8.24×10^−12^
HALLMARK_EPITHELIAL_MESENCHYMAL_TRANSITION	−0.567963778	−7.027159618	2.11×10^−12^	1.76×10^−11^
HALLMARK_COMPLEMENT	−0.582111115	−6.1058472	1.02×10^−9^	7.30×10^−9^
HALLMARK_IL2_STAT5_SIGNALING	−0.573317571	−5.768325086	8.01×10^−9^	5×10^−8^
HALLMARK_APICAL_JUNCTION	−0.540791657	−5.611683099	2.00×10^−8^	1.11×10^−7^
HALLMARK_P53_PATHWAY	−0.480027635	−5.561511073	2.67×10^−8^	1.34×10^−7^
HALLMARK_ESTROGEN_RESPONSE_LATE	−0.464291062	−5.123247455	3.00×10^−7^	1.25×10^−6^
HALLMARK_GLYCOLYSIS	−0.466279034	−5.039067076	4.68×10^−7^	1.80×10^−6^
HALLMARK_ALLOGRAFT_REJECTION	−0.497074315	−4.905992143	9.3×10^−7^	3.32×10^−6^
HALLMARK_KRAS_SIGNALING_UP	−0.573985774	−4.409147761	1.04×10^−5^	3.46×10^−5^
HALLMARK_ESTROGEN_RESPONSE_EARLY	−0.428017248	−4.001047521	6.31×10^−5^	0.000197071
HALLMARK_APOPTOSIS	−0.386057656	−3.582960349	0.000339722	0.000999183
HALLMARK_UV_RESPONSE_UP	−0.377520025	−3.377546754	0.000731355	0.002031542
HALLMARK_UV_RESPONSE_DN	−0.49585039	−3.229595402	0.001239655	0.00326225
HALLMARK_IL6_JAK_STAT3_Signaling	−0.515307214	−2.744010577	0.006069358	0.014450853

ANGPTL4, angiopoietin-like 4; ES, enrichment score; FDR, false discovery rate; NES, normalized enrichment score.

**Table VI.** Significant altered gene expression in low-angiopoietin-like 4 group based on The Cancer Genome Atlas data.

	logFold Change	P-value	Adjusted P-value
*CDH3*	−1.21550025	1.89×10^−5^	0.030221783
*NDRG1*	−1.156636514	3.76×10^−6^	0.009006122

## Data Availability

The data generated in the present study may be requested from the corresponding author.

## Abbreviations

ANGPTL4: angiopoietin-like 4
CI: confidence interval
DEG: differentially expressed gene
DFS: disease-free survival
FDR: false discovery rate
GSEA: gene set enrichment analysis
HNSCC: head and neck squamous cell carcinoma
HPV: human papillomavirus
HR: hazard ratio
OPSCC: oropharyngeal squamous cell carcinoma
OS: overall survival
PBS: phosphate-buffered saline
PCR: polymerase chain reaction
TCGA: The Cancer Genome Atlas
