# Supplementary material for: Inhibitory role of angiopoietin-like 4 for cancer progression in oropharyngeal squamous cell carcinoma
Source: Oncol Rep. 2026 Apr 20;55(6):117. doi: 10.3892/or.2026.9122 (PMC13122131; doi:10.3892/or.2026.9122)

Figure S1. Receiver operating characteristic curve analysis for angiopoietin-like 4 expression. The optimal cut-off value (7.7%) was determined by maximizing Youden's index.

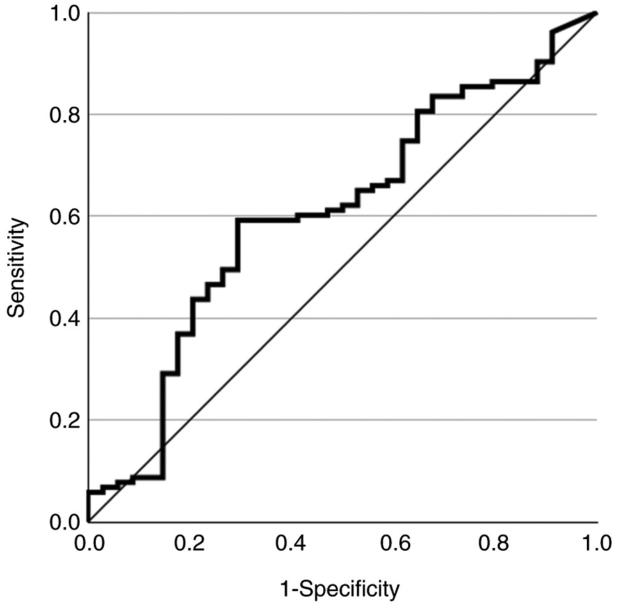

Supplement: Supporting Data [file Supplementary_Data1.pdf]
