# Supplementary material for: Inhibitory role of angiopoietin-like 4 for cancer progression in oropharyngeal squamous cell carcinoma
Source: Oncol Rep. 2026 Apr 20;55(6):117. doi: 10.3892/or.2026.9122 (PMC13122131; doi:10.3892/or.2026.9122)
Supplement: Supporting Data [file Supplementary_Data2.pdf]

Table SI. Sensitivity analyses of ANGPTL4 cut-off values for OS and DFS.

Cut-off values of ANGPTL4	Cases	5-year OS (%)	P-value	5-year DFS (%)	P-value
≥7.7%	71	88.4%	0.002	82.7%	<0.001
<7.7%	66	61.6%		52.5%	
≥median (8.8%)	69	85.5%	0.003	81.2%	<0.001
<median (8.8%)	68	64.7%		54.4%	
≥10%	66	84.8%	0.013	80.3%	0.004
<10%	71	66.2%		56.3%	
≥15%	62	83.9%	0.045	80.6%	0.007
<15%	75	68.0%		57.3%	

ANGPTL4, angiopoietin-like 4; DFS, disease-free survival; OS, overall survival.

Table SII. Patients' characteristics stratified by p16 status.

Variables	p16-positive	p16-negative	P-value
Overall	109	28	
Age			0.189
<65	58	11	
≥65	51	17	
Sex			0.333
Male	91	25	
Female	18	3	
Subsite			0.717
Anterior wall	31	7	
Others	78	21	
T classification			0.935
1, 2	77	20	
3,4	32	8	
N classification			0.025
0	24	12	
1,2,3	85	16	
M classification			0.632
0	107	28	
1	2	0	
TNM stage			<0.001
I, II	87	11	
III, IV	22	17	
ANGPTL4			0.828
≥7.7%	57	14	
<7.7%	52	14	

ANGPTL4, angiopoietin-like 4.

Table SIII. Prognostic impact of ANGPTL4 expression stratified by p16 status.

	ANGPTL4 expression	Cases	5-year OS (%)	P-value	5-year DFS (%)	P-value
p16-positive	≥7.7%	57	87.7%	0.001	82.5%	0.002
	<7.7%	52	59.6%		53.8%	
p16-negative	≥7.7%	14	78.6%	0.796	78.6%	0.115
	<7.7%	14	78.6%		50.0%	

ANGPTL4, angiopoietin-like 4; DFS, disease free survival; OS, overall survival.

Table SIV. Multivariate analysis of prognostic factors for OS and DFS in patients underwent definitive therapy: Additional adjustment for p16 status and initial definitive therapy.

Variables	Model 1 (Significant variables in Table II + p16 status)						Model 2 (Model 1 + initial definitive therapy)					
	OS			DFS			OS			DFS		
	HR	95% CI	P-value	HR	95% CI	P-value	HR	95% CI	P-value	HR	95% CI	P-value
Subsite (Anterior wall)	-	-	-	1.806	0.900-3.624	0.096	-	-	-	1.806	0.899-3.629	0.097
T classification (3, 4)	2.86	1.120-7.301	0.028	3.300	1.447-7.525	0.005	2.860	1.120-7.301	0.028	3.302	1.440-7.571	0.005
TNM stage (III, IV)	1.029	0.360-2.941	0.957	0.874	0.340-2.249	0.781	1.029	0.360-2.941	1.029	0.875	0.340-2.252	0.781
p16 status (positive)	1.128	0.404-3.150	0.819	0.809	0.350-1.870	0.620	1.128	0.404-3.150	0.819	0.809	0.350-1.871	0.620
Initial definitive therapy (surgery)	-	-	-	-	-	-	1.378	0.628-3.025	0.424	1.004	0.512-1.969	0.990
ANGPTL4 (<7.7%)	3.656	1.556-8.588	0.003	4.012	1.847-8.715	<0.001	3.656	1.556-8.588	0.003	4.014	1.842-8.746	<0.001

Models were restricted to patients who received definitive therapy. Initial definitive therapy was categorized as surgery vs. (chemo)radiation. Because no M1 patients were present in this subset, M classification was not included in these models. ANGPTL4, angiopoietin-like 4; CI, Confidence interval; DFS, Disease free survival; HR, hazard ratio; OS, overall survival.
